# Cisplatin generates oxidative stress which is accompanied by rapid shifts in central carbon metabolism

**DOI:** 10.1038/s41598-018-22640-y

**Published:** 2018-03-09

**Authors:** Wangie Yu, Yunyun Chen, Julien Dubrulle, Fabio Stossi, Vasanta Putluri, Arun Sreekumar, Nagireddy Putluri, Dodge Baluya, Stephen Y. Lai, Vlad C. Sandulache

**Affiliations:** 1 Bobby R. Alford Department of Otolaryngology Head and Neck Surgery, Baylor College of Medicine, Houston, TX USA; 2 Department of Head and Neck Surgery, University of Texas MD Anderson Cancer Center, Houston, TX USA; 3 Department of Molecular and Cellular Biology, Baylor College of Medicine, Houston, TX USA; 4 Advanced Technology Core, Dan L. Duncan Comprehensive Cancer Center, Baylor College of Medicine, Houston, TX USA; 5 Chemical Imaging Research Core, University of Texas MD Anderson Cancer Center, Houston, TX USA; 6 Integrated Microscopy Core, Advanced Technology Cores, Baylor College of Medicine, Houston, TX USA; 7 Department of Molecular and Cellular Oncology, University of Texas MD Anderson Cancer Center, Houston, TX USA

## Abstract

Cisplatin is commonly utilized in the treatment of solid tumors. Its mechanism of action is complex and multiple mechanisms of resistance have been described. We sought to determine the impact of cisplatin-generated oxidative stress on head and neck squamous cell carcinoma (HNSCC) proliferation, survival and metabolic activity in order to identify a potential metabolic signature associated with cisplatin response. DNA-bound cisplatin represents a small fraction of total intra-cellular cisplatin but generates a robust oxidative stress response. Neutralization of oxidative stress reverses cisplatin toxicity independent of the mechanism of cell death and *TP53* mutational status. Cisplatin-induced oxidative stress triggers rapid shifts in carbon flux in 3 commonly utilized catabolic pathways: glycolysis, pentose phosphate pathway and citric acid cycle. Among these metabolic shifts, decreased flux from pyruvate into lactate is the only metabolic effect consistently observed across multiple HNSCC cell lines of varying genomic backgrounds and may reflect differential cisplatin sensitivity. Oxidative stress is a critical component of cisplatin cytotoxicity in HNSCC and is reflected in acute changes in carbon flux from pyruvate into lactate. This suggests that lactate may contribute to a metabolic signature of acute cisplatin toxicity, and could prove useful in optimizing cisplatin-based treatment regimens in HNSCC.

## Introduction

Cisplatin is one of the most commonly utilized anti-neoplastic agents in multiple solid tumors including ovarian carcinoma, non-small cell lung carcinoma and head and neck squamous cell carcinoma (HNSCC)^1–3^. In HNSCC, a deadly disease which affects 50,000 new individuals in the US each year, cisplatin resistance is associated with decreased survival^4^. Multiple mechanisms of cisplatin resistance have been described in HNSCC and other solid tumors in part due to its complex mechanism of action^5–9^. It is therefore critical to identify: 1) the primary mechanism of cisplatin activity in specific tumor types (i.e. HNSCC) and 2) potential adaptive mechanisms contributing to differential cisplatin response.

Traditionally, cisplatin toxicity has been described as a function of DNA binding, followed by single-stranded DNA breaks^7^. More recently, cisplatin has been shown to generate oxidative stress which can also contribute to its anti-tumor effects^7,10–14^. The impact of cisplatin-induced oxidative stress in HNSCC remains poorly understood in large part because cisplatin effects on HNSCC vary not only quantitatively (resistant *vs* sensitive), but also qualitatively (i.e. senescence vs mitotic catastrophe)^8,15^. Oxidative stress has been shown by us and others to generate metabolic perturbations in HNSCC and other tumors types^16–18^. Although HNSCC tumor metabolism can vary to some degree based on the underlying genomic background, we previously showed that HNSCC cell lines are predominantly dependent on glucose catabolism and have a high glycolytic rate^18^.

We have demonstrated that radiation-induced oxidative stress can perturb the cellular redox state (ratio of reducing to oxidizing equivalents) and generate transient perturbations in carbon flux^16,17,19^. In the current study we sought to determine: 1) the relative contribution of oxidative stress to cisplatin effects in HNSCC and 2) the impact of cisplatin-generated oxidative stress on HNSCC carbon flux. Identification of metabolic pathways altered in response to cisplatin could not only generate putative biomarkers of cisplatin response, but also identify potentially actionable methods of cisplatin sensitization.

## Materials and Methods

### Cells

Previously described cell lines (head and neck squamous cell carcinoma-HNSCC) were obtained from an established cell line bank in the laboratory of Dr. Jeffrey N. Myers under approved institutional protocols. All cell lines were routinely tested and authenticated using short tandem repeat analysis every 3 months^20^. Cells were maintained in either RPMI or MEM growth media supplemented with glutamine, pyruvate, penicillin/streptomycin and 10% fetal bovine serum.

### Chemicals and antibodies

N-acetyl cysteine (NAC), oxamate and cisplatin were purchased from Sigma-Aldrich (MO, USA); for animal experiments cisplatin was obtained from Teva Pharmaceutical (NC, USA). The following antibodies were used in this study: anti-p53 DO-1(Santa Cruz Biotechnology, CA, USA), anti-phospho-P53 (Cell Signaling, MA, USA), anti-p21 (EMD Millipore, Billerica, MA, USA), p-histone γ H2AX S139 (Cell signaling, MA, USA) and $\beta$-actin (Sigma, MO, USA).

### Redox, ATP and lactate biochemical studies

For lactate, LDH activity, NAD+/NADH and NADP+/NADPH measurements, cells were harvested at various time points following drug treatment using appropriate buffers and frozen in liquid nitrogen. Intra-cellular lactate, NAD+, NADH, NADP+ and NADPH levels along with *ex vivo* LDH activity were analyzed using commercially available colorimetric assays (BioVision, CA, USA), according to the manufacturer’s instructions as previously described by our group and others^17,19^. Intra-cellular ATP levels were measured using commercially available luminescent assays (Promega, WI, USA) as previously described by our group and others; ATP levels were normalized to cell number quantified using Hoechst staining as previously described by our group^18^.

### Cytotoxicity studies

Drug cytotoxicity was assayed using either clonogenic assays or using total DNA content as a surrogate for cell number^21^. For clonogenic assays, cells were treated with the indicated drug for 24 hours. Fresh media were replaced and cells were incubated for colony formation for 10–14 days, then fixed and stained using a 0.05% crystal violet in 10% formalin solution. Colonies were counted and surviving fractions were determined based upon the plating efficiency of the control group. Drug effects were also evaluated using a more conventional 72 hour assay. Briefly, cells were seeded in 96-well plates and exposed to various drug concentrations. Drug effects were ascertained 72 hours later using total DNA content (Hoechst staining) as a surrogate for cell number^21^. Cell cycle analysis was performed using propidium iodide labeling of cells at various time points following drug exposure^18^.

### Senescence

Senescence-associated (SA)-β-gal staining was carried out according to the manufacturer’s instructions (Cell Signaling, MA, USA). Briefly, HNSCC cells plated at sub-confluence were exposed to various agents for 24 hours. The media was then replaced with fresh media and cells were maintained in culture for 5 days. Cells were then fixed for 10 minutes and stained overnight for SA-β-gal activity at 37 °C. Blue-staining cells were imaged on a Biotek Cytation5 instrument, manually scored as senescent and reported as a percentage of all the cells observed per high power field.

### Western blotting

Cells were lysed in radioimmunoprecipitation assay (RIPA) buffer (20 mM Tris at pH 7.5, 150 mM NaCl, 1 mM EDTA, 1 mM EGTA, 1% NP-40, 1% sodium deoxycholate) containing 1 mM PMSF (phenylmethylsulfonyl fluoride), 1 mM DTT, 5 μg/ml aprotinin, 10 μg/ml leupeptin, 1 μg/ml pepstatin A, 1 mM Na3VO4, 1 mM NaF, 1 mM β-glycerophosphate, and 2.5 mM sodium pyrophosphate. The lysate was sonicated and then centrifuged for 10 min. Equal amounts of protein extract were loaded, transferred, and probed with indicated antibodies. Immunoblots were visualized using Clarity Western ECL (Bio-Rad, CA, USA).

### Cisplatin measurements

Cells were exposed to cisplatin for varying periods of time in the presence or absence of other compounds. For whole-cell extract samples, cells were washed twice with PBS; 250 µl of aqua regia was added to each sample. Cell suspensions were then incubated for 1–2 hours followed by the addition of 5 mL of deionized water. For DNA-bound extract samples, treated cells were washed twice with PBS prior to addition of and DNAzol reagent (Life Technologes, Warrington, UK). The final DNA pellet of each sample was suspended in 150 µl of 8 mM NaOH. The DNA solution was then solubilized in 500 μl aqua regia for 1–2 hours followed by the addition of 5 mL of deionized water. Cell extracts were filtered using a 0.22 μm filter system before platinum concentration were measured by ICP-MS. Platinum (Pt) levels were measured using an Agilent 7900 ICP-MS coupled to an SPS 4 Autosampler (SantaClara, CA, USA). The standards used for Pt calibrations and internal standard (Iridium- Ir) were obtained from Inorganic Ventures (Christiansburg, VA, USA). Solvents and acids (water, hydrochloric acid and nitric acid) were ICP-MS grade and obtained from Fisher Scientific (Hampton, NH, USA). For detection, masses at 194, 195 and 196 for Pt and 193 for Ir were monitored and integration time was 0.1 s. For each sampling, a rinse cycle of acid and water wash was included. Calibration solution concentrations were made as follows: 0.125, 1.25, 12.5, 25 and 50 parts per billion (ppb) using serial dilutions. Calibration curve was made using the signal ratios of Pt to Ir with weighted correction using 1/x^2^.

### Gamma-H2AX immunofluorescence, imaging and image analysis

Cells were plated in 384-well glass bottom plates (Greiner Bio-One, NC, USA), treated with various drugs, fixed in 4% paraformaldehyde for 20 minutes on ice, quenched with ammonium chloride 0.1 M in PBS for 15 minutes and permeabilized with PBS/Triton-X100 0.1% for 20 minutes at room temperature. After 30 minutes of blocking in 5% milk/TBST, cells were incubated at 4 °C with anti-phospho-γH2Ax rabbit polyclonal antibody at 1/500 (Cell Signaling Technology, MA, USA). After washing, cells were incubated with Alexa488-conjugated anti-rabbit antibody (1/1000, Cell Signaling Technology, MA, USA) at room temperature and counterstained with DAPI (2 µg/ml). Plates were imaged on an IC200 (VALA Sciences, CA, USA) high-throughput microscope using a Nikon Plan Apo 20x/0.95 objective, 4 fields of view per well, one z-plane. Image analysis was performed using MyImageAnalysis, a web-based, PipelinePilot (Biovia)-powered image analysis application^22^. Briefly, nuclei were segmented in the DAPI channel using a marker-based watershed algorithm. The nuclear segmentation was then used as a mask to extract mean pixel intensity values in the γH2Ax channel for each cell. Cells were considered γH2Ax positive when their mean pixel intensity was higher than the average pixel intensity plus one standard deviation of DMSO-treated cells. Between 35,000 and 72,000 cells were analyzed per condition. Graphs were generated in GraphPad Prism v.5.0. To analyze the number of γH2Ax foci per nucleus and to show representative images at higher magnification, cells were imaged with a GE Healthcare DeltaVision deconvolution microscope, using an Olympus 40x/0.95NA PlanApo objective. Z-stacks (0.35 µm steps) were acquired, covering the entire nucleus, and max projected after restorative deconvolution. Foci were identified using a local maxima detection algorithm developed in MatLab, and the number of foci per cell, their sizes and intensities were extracted.

### Isotope labeling and profiling by targeted MS

Nutrients labeled with ^13^C were purchased from Cambridge Isotope Laboratories. Cells were grown in regular media until 60% confluence and then exposed to test conditions, followed by addition of 10 mM D-glucose [U-^13^C_6_] glucose in growth media supplemented with 10%FBS, non-essential amino acids, glutamine and pyruvate. Cells from each treatment were frozen using liquid nitrogen. Cells were lysed using a 0.5-ml mixture of 1:1 water/methanol, sonicated for 1 minute (two 30-second pulses), and then mixed with 450 μl ice-cold chloroform. The resulting homogenate was then mixed with 150 μl ice-cold water and vortexed again for 2 minutes. The homogenate was incubated at −20 °C for 20 minutes and centrifuged at 4 °C for 10 minutes to partition the aqueous and organic layers. The aqueous and organic layers were combined and dried at 37 °C for 45 minutes in an automatic Environmental Speed Vac system (Thermo Fisher Scientific). The extract was reconstituted in a 500-μl solution of ice-cold methanol/water (1:1) and filtered through a 3-kDa molecular filter (Amicon Ultracel 3-kDa Membrane) at 4 °C for 90 minutes to remove proteins. The filtrate was dried at 37 °C for 45 minutes in a speed vacuum and stored at −80 °C until MS analysis. Prior to MS analysis, the dried extract was resuspended in a 50-μl solution of methanol/water (1:1) containing 0.1% formic acid and then analyzed using multiple reaction monitoring (MRM). Ten microliters were injected and analyzed using a 6490 QQQ triple quadrupole mass spectrometer (Agilent Technologies) coupled to a 1290 Series HPLC system via selected reaction monitoring (SRM). Metabolites were targeted in both positive and negative ion modes: the electrospray source ionization (ESI) voltage was +4,000 V in positive ion mode and −3,500 V in negative ion mode. Approximately 9 to 12 data points were acquired per detected metabolite. To target the TCA flux, the samples were delivered to the mass spectrometer via normal-phase chromatography using a Luna Amino column (4 μm, 100 A 2.1 × 150 mm). For ^13^C-labeled experiments, SRM was performed for expected ^13^C incorporation in various forms for targeted LC-MS/MS. Mass isotopomer distribution (MID) was calculated using the formula: [fractional incorporation = (^13^C/^13^C + ^12^C) × 100] and corrected for natural abundance. The change in reductive carboxylation flux was calculated by comparing the MIDs of TCA metabolites from [U-^13^C_6_]Glucose–labeled cells.

### HNSCC tumors in mice

Female athymic nude mice (8–12 weeks) (Envigo, Indianapolis, IN) were maintained in a pathogen-free facility and fed irradiated mouse chow and autoclaved, reverse osmosis treated water. The animal facility was approved by the American Association for the Accreditation of Laboratory Animal Care and met all current regulations and standards of the U.S. Department of Agriculture, U.S. Department of Health and Human Services and the National Institutes of Health. All procedures were approved by the Institutional Animal Care and Use Committee of The University of Texas MD Anderson Cancer Center. For flank tumors, cells (2 × 10^6^/mouse) were injected into both flanks of each animal. Tumor size was ascertained regularly throughout the experimental period using manual measurements as previously described^16,23^. Tumors were allowed to grow for ~1 week prior to initiation of experiments. Mice received a single dose of cisplatin administered via tail vein injection (2 mg/kg). Following completion of experiments, tumors were harvested for either biochemical analysis or histologic and immunohistochemical analysis. The number of animals chosen for each *in vivo* experiment was based on previous experience with this animal model and the expected effect size. Statistical analysis of *in vivo* data was conducted as described below.

### Statistical analysis

All *in vitro* experiments were carried out at least in triplicate (for each condition) and were repeated to ensure reproducibility. All statistical analysis for *in vitro* and *in vivo* experiments was conducted using two-tailed, Student’s t-test analysis with a cutoff p-value of 0.05 to demonstrate statistical significance.

### Data availability

The datasets generated during and/or analyzed during the current study are available from the corresponding author on reasonable request.

## Results

### Cisplatin inhibits HNSCC proliferation and survival

**Figure 1:**
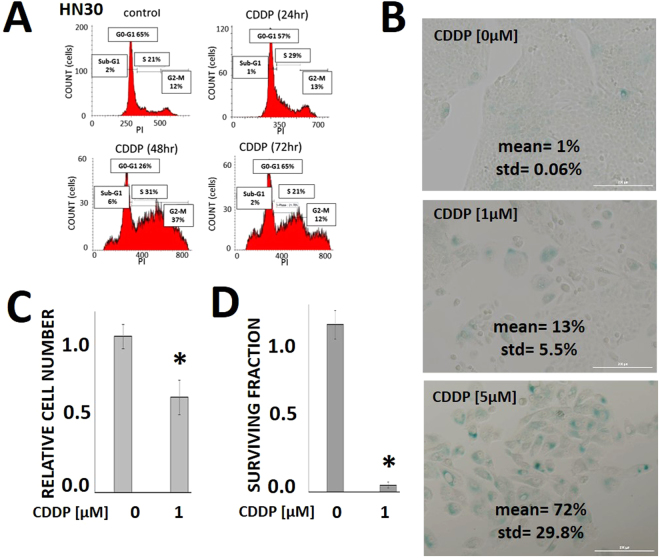
Cisplatin triggers senescence and decreases proliferation. (**A**) HN30 cells were exposed to CDDP [5 µM] for 24–72 hours and stained with propidium iodide (PI). Flow cytometry was used to ascertain cellular fraction in each phase of the cell cycle. (**B**) Cells were grown at sub-confluence, treated with CDDP for 24 hours and then allowed to recover for 5 days. After 5 days, the cells were fixed and stained for beta-galactosidase activity. Senescence data are expressed as mean fraction of beta-galactosidase positive cells for each condition. Each experiment was carried out in triplicate (std = standard deviation). (**C**) Cells were exposed to CDDP for 48 hours. Relative cell number was measured using the Hoechst DNA assay and compared to the control condition. Error bars represent standard deviation; *indicates p < 0.05. Each experiment was carried out in triplicate. (**D**) Cells were exposed to CDDP for 24 hours and allowed to form colonies. Surviving fraction was ascertained 10–14 days later and expressed as a function of the control condition. Error bars represent standard deviation; *indicates p < 0.05.

Since cell response to CDDP has been shown to be partially dependent on the cellular genomic background, we conducted the initial analysis using the HN30 cell line, an established, previously described and validated cell line which expresses wild-type, functional *TP53*^15,18,24^. In this cell line, CDDP does not generate significant levels of apoptosis (Fig. 1A) but triggers senescence, resulting in decreased proliferation and clonogenic survival (Fig. 1B–D).

### Cisplatin-generated oxidative stress is critical to effects on HNSCC proliferation

**Figure 2:**
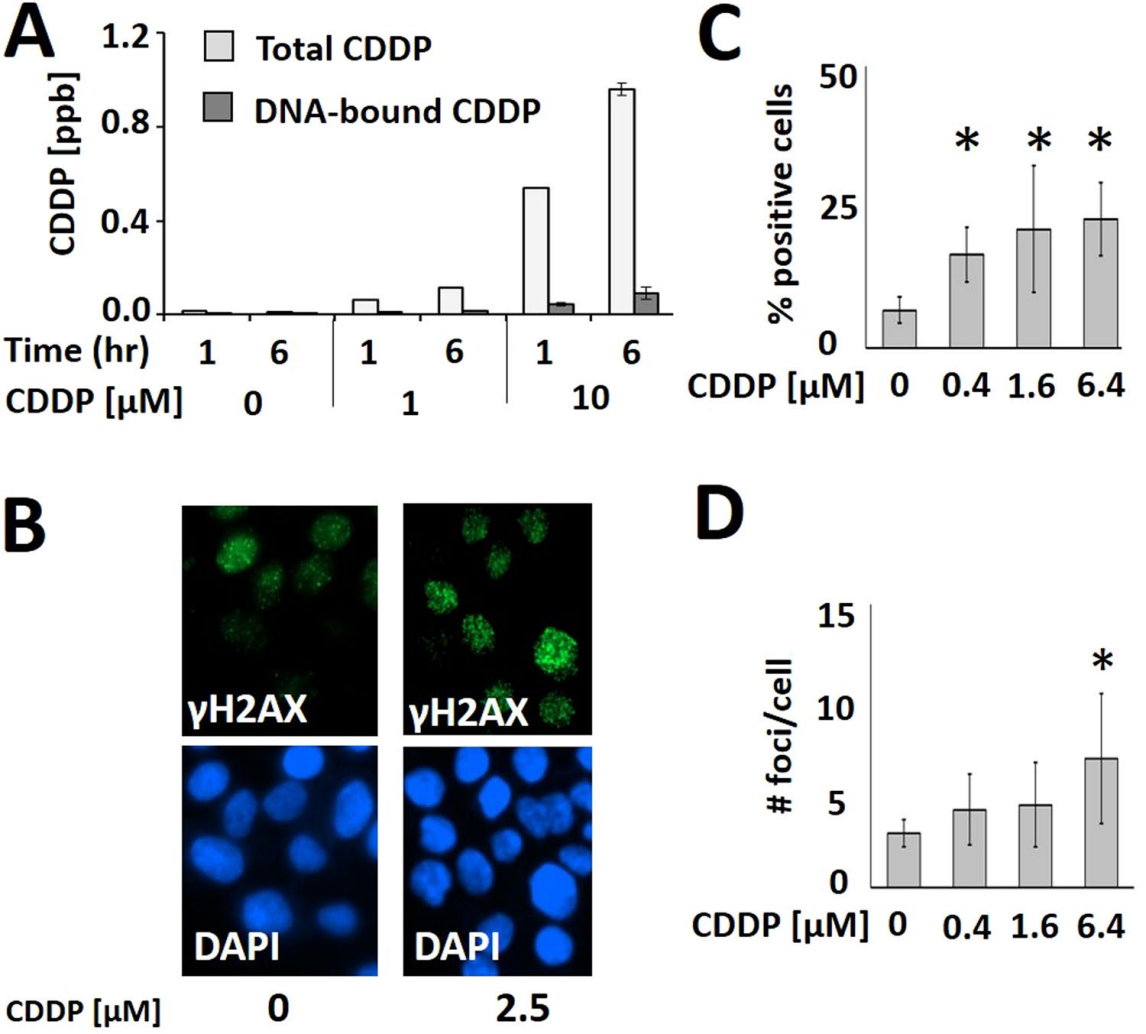
Cisplatin generates oxidative stress in HNSCC. (**A**) Following CDDP treatment total cell extracts and the DNA-bound cellular fraction were subjected to ICP-MS for quantitative measurement of the CDDP concentration (ppb = parts per billion). (**B**–**D**) Cells were exposed to CDDP for 16 hours [2.5 µM], fixed and immunolabeled with phospho-γH2AX antibody. Using high throughput microscopy and single cell image analysis, two parameters were evaluated as a function of CDDP concentration: (**C**) % positive cells (nuclear intensity > mean +1 standard deviation of negative controls) and (**D**) number of foci/cell. *Indicates p < 0.05 compared to control condition.

**Figure 3:**
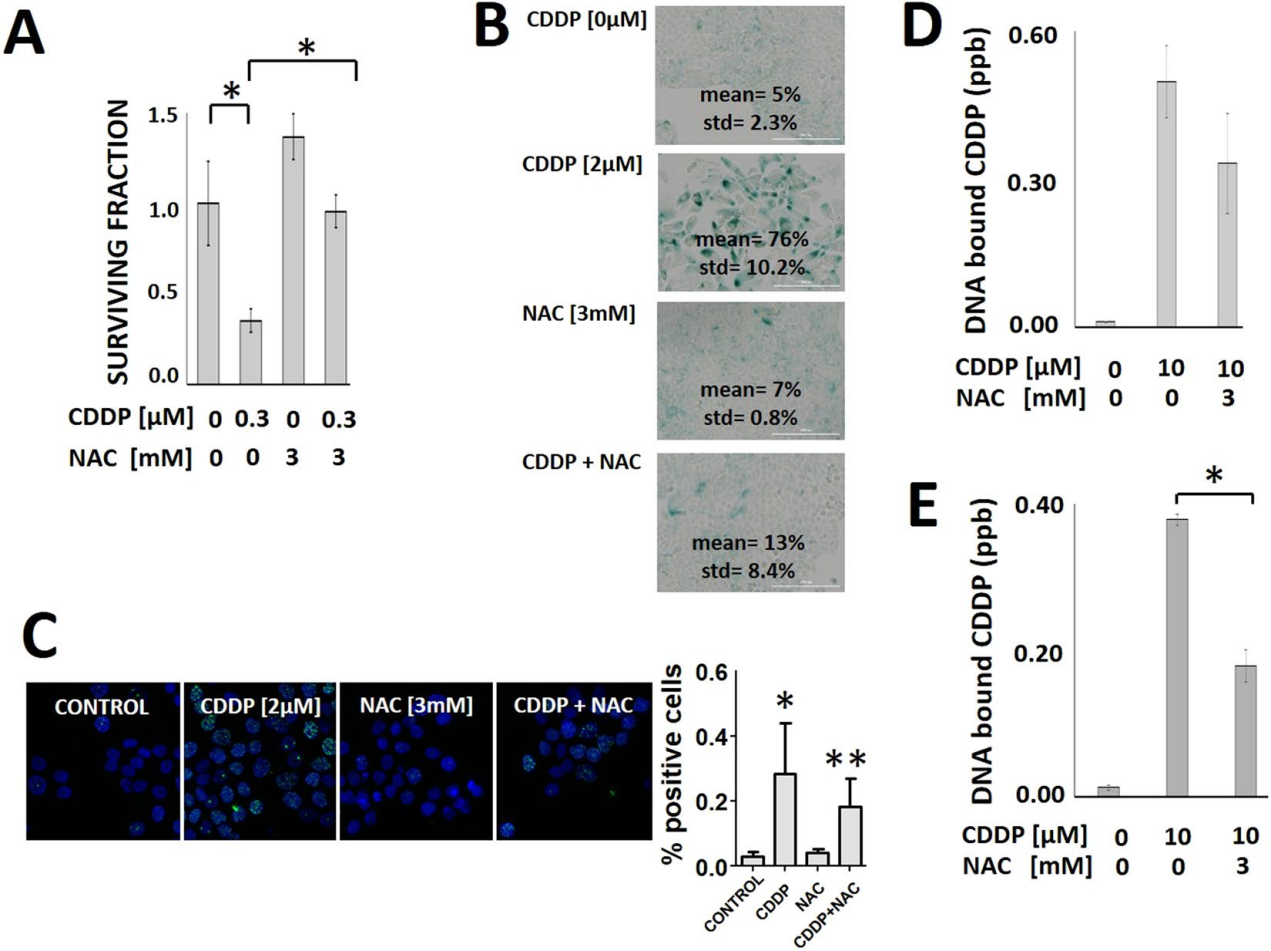
Oxidative stress contributes to cisplatin toxicity in HNSCC. Cells were exposed to CDDP following a 1 hour pre-treatment with NAC and evaluated using: (**A**) clonogenic survival assay, (**B**) senescence (*Indicates p < 0.05, std = standard deviation) and (**C**) γH2AX foci analysis (*Indicates p < 0.05 compared to control condition; **Indicates p < 0.05 compared to CDDP only condition). NAC reversed CDDP effects in all 3 assays. Cells were pre-treated with NAC for 1 hour (**D**) or 24 hours (**E**). DNA-bound CDDP was then measured using ICP-MS. *Indicates p < 0.05. All conditions were tested at least in duplicate and each experiment was repeated at least twice. Data are presented as means with error bars representing standard deviation.

Measurements of total and DNA-bound CDDP indicate that only a small fraction (<10%) of CDDP is DNA-bound (Fig. 2A). CDDP generates a significant, dose-dependent increase in γH2AX foci formation (Fig. 2B–D), suggesting a significant increase in cellular oxidative stress. To determine whether oxidative stress is a significant contributor to DNA effects in HNSCC we evaluated the effects of the free radical scavenger N-acetyl cysteine (NAC) on CDDP toxicity. NAC reversed CDDP toxicity, decreased senescence and reduced γH2AX foci formation (Fig. 3A–C). NAC had a modest effect on DNA-bound CDDP levels (Fig. 3D) only following extended pre-exposure to the drug (24 hours) (Fig. 3E).

### Cisplatin-generated oxidative stress impacts reducing potential and carbon flux

**Figure 4:**
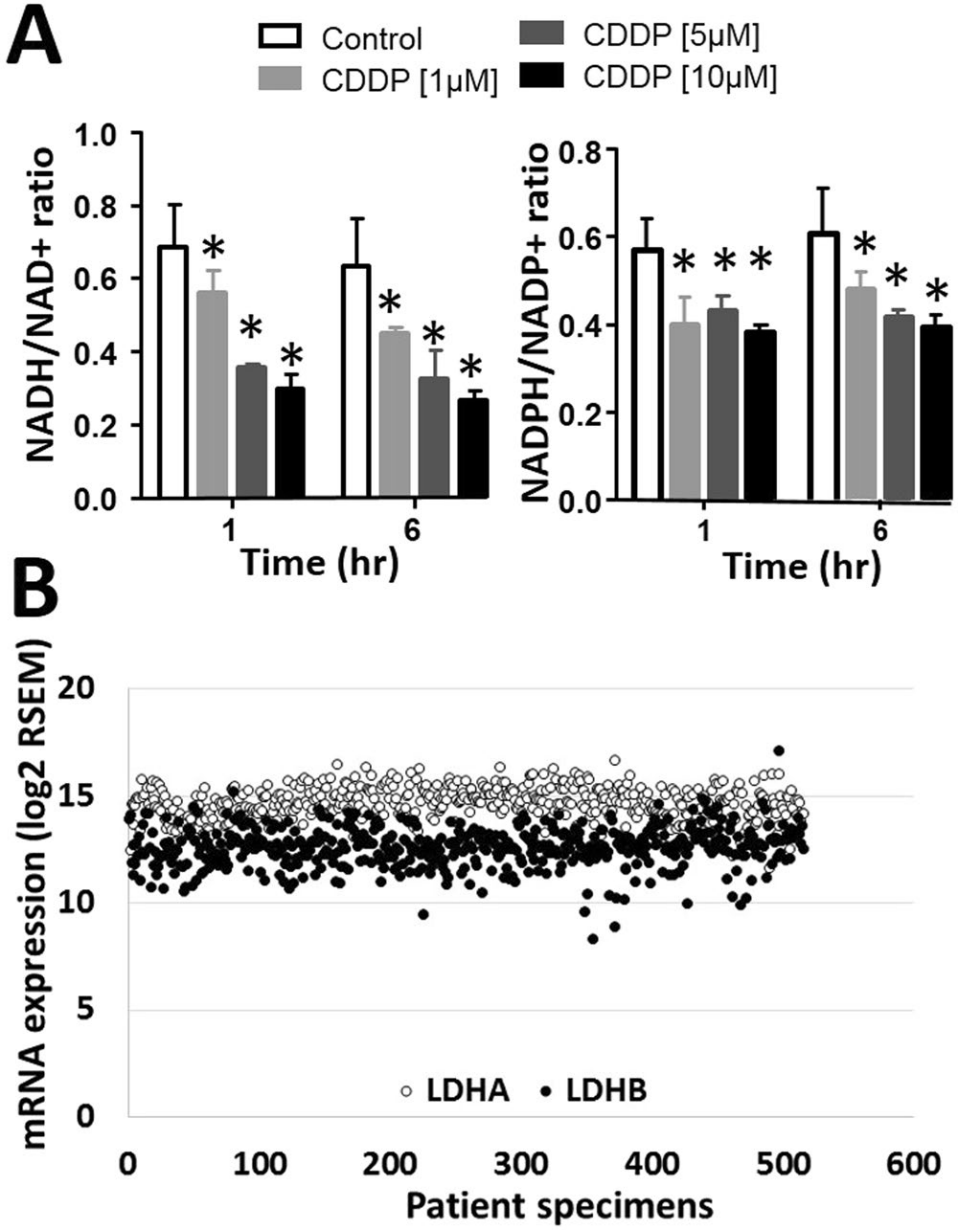
Cisplatin generated oxidative stress decreased reducing equivalent availability. (**A**) Cells were treated with CDDP at the indicated doses and cellular NADH, NAD+, NADPH and NADP+ levels were measured. (**B**) TCGA HNSCC tumors were evaluated for mRNA expression levels for LDHA and LDHB. Data are represented as log2 normalized RSEM gene expression values available from the HNSCC TCGA cohort (https://gdac.broadinstitute.org/, June 2017). *Indicates p < 0.05. All conditions were tested at least in triplicate and each experiment was repeated at least three times. Data are presented as means with error bars representing standard deviation.

NADH and NADPH are the two most abundant reducing equivalents inside tumor cells. CDDP decreased both NADH/NAD+ and NADPH/NADP+ ratios in a dose-dependent manner in a rapid time frame (1–6 hours) (Fig. 4A), but did not decrease ATP levels over the same time frame (Supplemental Table 1). Reducing equivalents are essential for normal metabolic flux through a variety of basic metabolic pathways such as glycolysis, the pentose phosphate pathway (PPP) and the citric acid cycle (TCA). Because HNSCC tumors are predominantly glycolytic, the most likely metabolic pathway to be affected by redox fluctuations should be glycolysis, more specifically the conversion of pyruvate into lactate which requires NADH as a coenzyme^18^. Analysis of existing HNSCC TCGA data indicates quite clearly that LDHA and LDHB expression is high across patient tumors and confined to a relative narrow spectrum of values (Fig. 4B).

To evaluate CDDP-driven fluctuations in carbon metabolism, we focused on the 3 primary or central metabolic pathways that are critical for energy, biomass and reducing equivalent flux in tumor cells: glycolysis (including pyruvate to lactate conversion), the citric acid cycle (TCA) and the pentose phosphate pathway (PPP) (Supp. Figure 1). HNSCC cells take up ^13^C glucose and rapidly convert it into pyruvate and subsequently into labeled lactate. Since excess pyruvate is a normal media additive for HNSCC cells, this experiment was carried out in the presence of unlabeled pyruvate which explains the discrepancy in ^13^C label incorporation between pyruvate in lactate (90 vs 65%). In contrast, <20% of alpha-ketoglutarate contained ^13^C label and only 40–50% of the PPP intermediates ribose/ribulose/xylulose-5P and sedoheptulose-7P contained ^13^C label.

**Figure 5:**
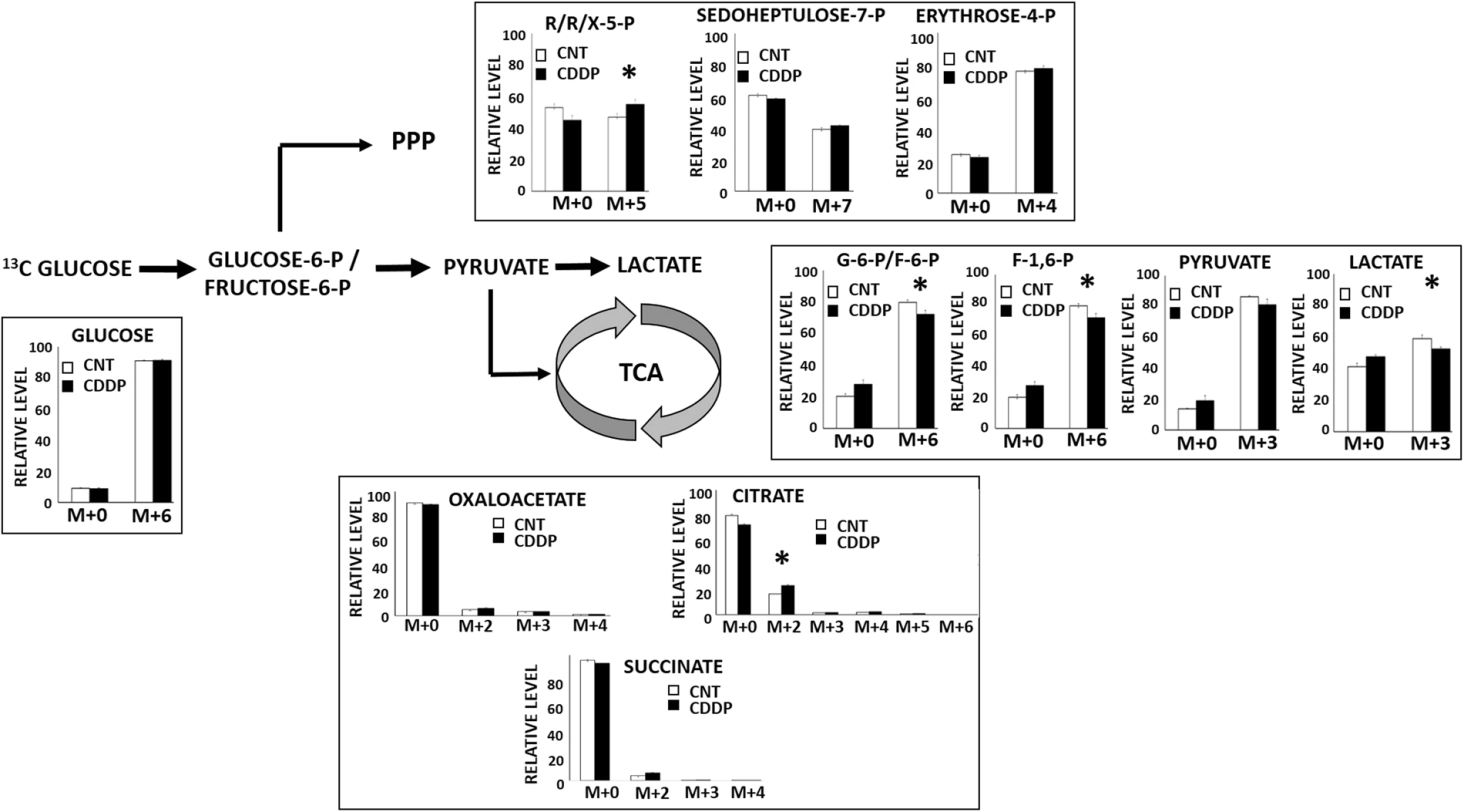
Cisplatin alters central carbon flux in HNSCC. HN30 cells were exposed to 10 mM all carbon (U) ^13^C-labeled glucose for 3 hours in the presence or absence of CDDP [10 µM]. Cells were harvested and metabolite levels (unlabeled- m + 0, labeled- m + (2–6)) were quantitatively measured. Each condition was tested in triplicate. Data are presented as means, with error bars indicating standard error of the mean. (R/R/X-5-P = ribose/ribulose/xylulose-5-phosphate, G-6-P = glucose-6-phosphate, F-6-P = fructose-6-phosphate, F-1,6-P = fructose 1,6 bisphosphate); m + X indicates mass shift of X from unlabeled glucose value. *Indicates change is statistically significant compared to the control condition, p-value < 0.05.

**Figure 6:**
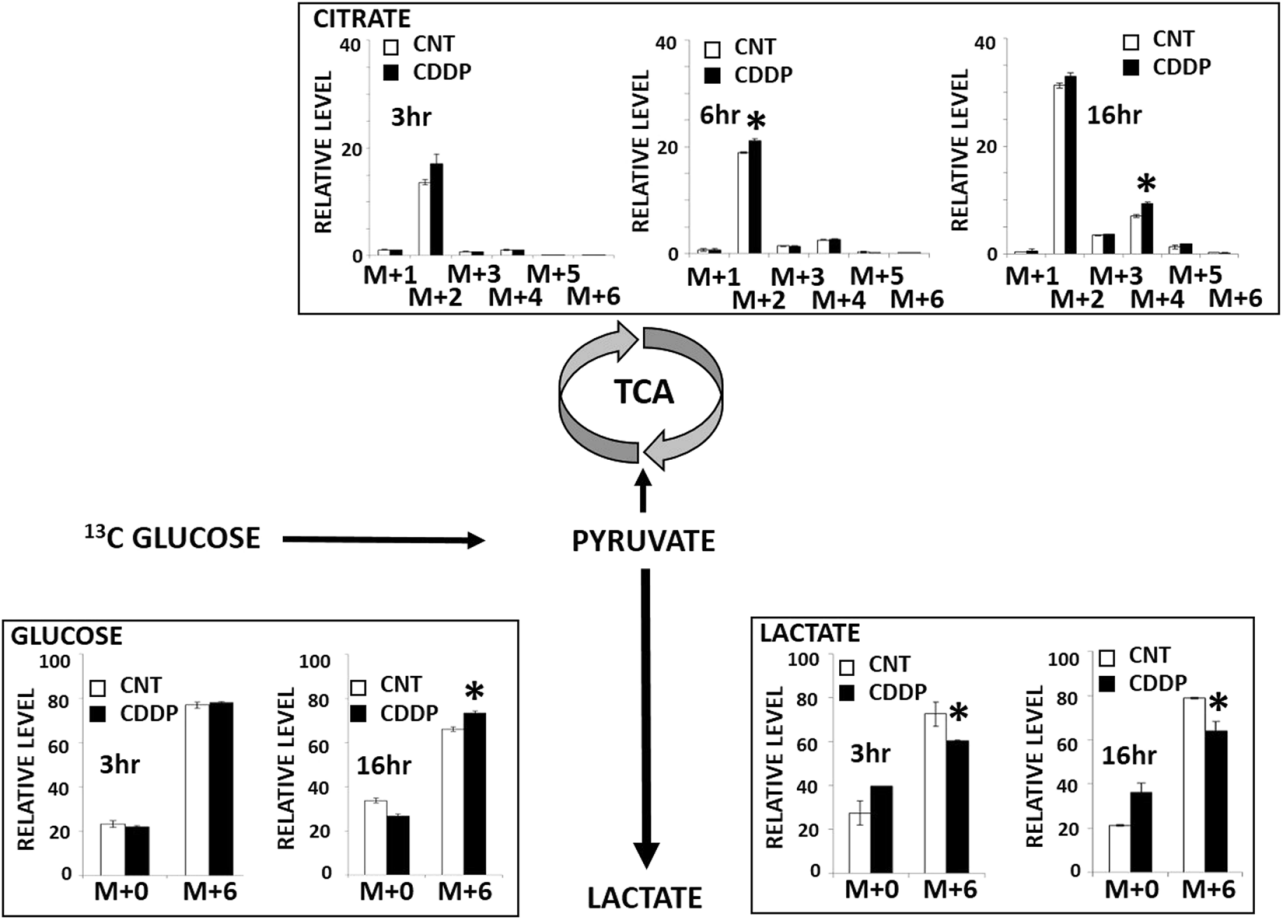
Cisplatin-induced changes in central carbon flux persist over time. HN30 cells were exposed to 10 mM all carbon (U) ^13^C-labeled glucose for 3 or 16 hours in the presence or absence of CDDP [5 µM]. Cells were harvested and metabolite levels (unlabeled- m + 0, labeled- m + (2–6)) were quantitatively measured. Each condition was tested in triplicate. Data are presented as means, with error bars indicating standard error of the mean; m + X indicates mass shift of X from unlabeled glucose value. *Indicates change is statistically significant compared to the control condition, p-value < 0.05.

Following acute (3 hours) exposure to CDDP, HN30 cells demonstrated preservation of ^13^C glucose uptake but decreased levels of ^13^C-labeled glycolytic intermediates including lactate, pyruvate, glucose-6-P/fructose-6-P and fructose-1,6-P (Fig. 5). Cellular levels of 3-carbon intermediates were preserved (data not shown), with the exception of lactate and pyruvate. A statistically significant compensatory increase in labeled citrate was measured in the presence of CDDP and a similar increase in labeled ribose/ribulose/xylulose-5P suggesting a potential shunting into TCA and PPP metabolic activity due to decreased carbon flux through the pyruvate to lactate reaction. At 16 hours following CDDP exposure decreased lactate generation is preserved (Fig. 6) but compensatory changes became more evident with increased shunting and incorporation of ^13^C label into citrate. Increased incorporation of ^13^C label into the M + 2 and M + 4 moieties over time was consistent with the cyclical nature of carbon incorporation into TCA intermediates. Importantly, relative glucose uptake at this time point actually increased suggesting a cellular attempt to overcome the cisplatin-induced metabolic stress. These experiments were carried out in the absence of unlabeled pyruvate in order to maximize ^13^C label incorporation into lactate and at a lower cisplatin concentration [5 µM] due to the extended time frame of the experiment.

**Figure 7:**
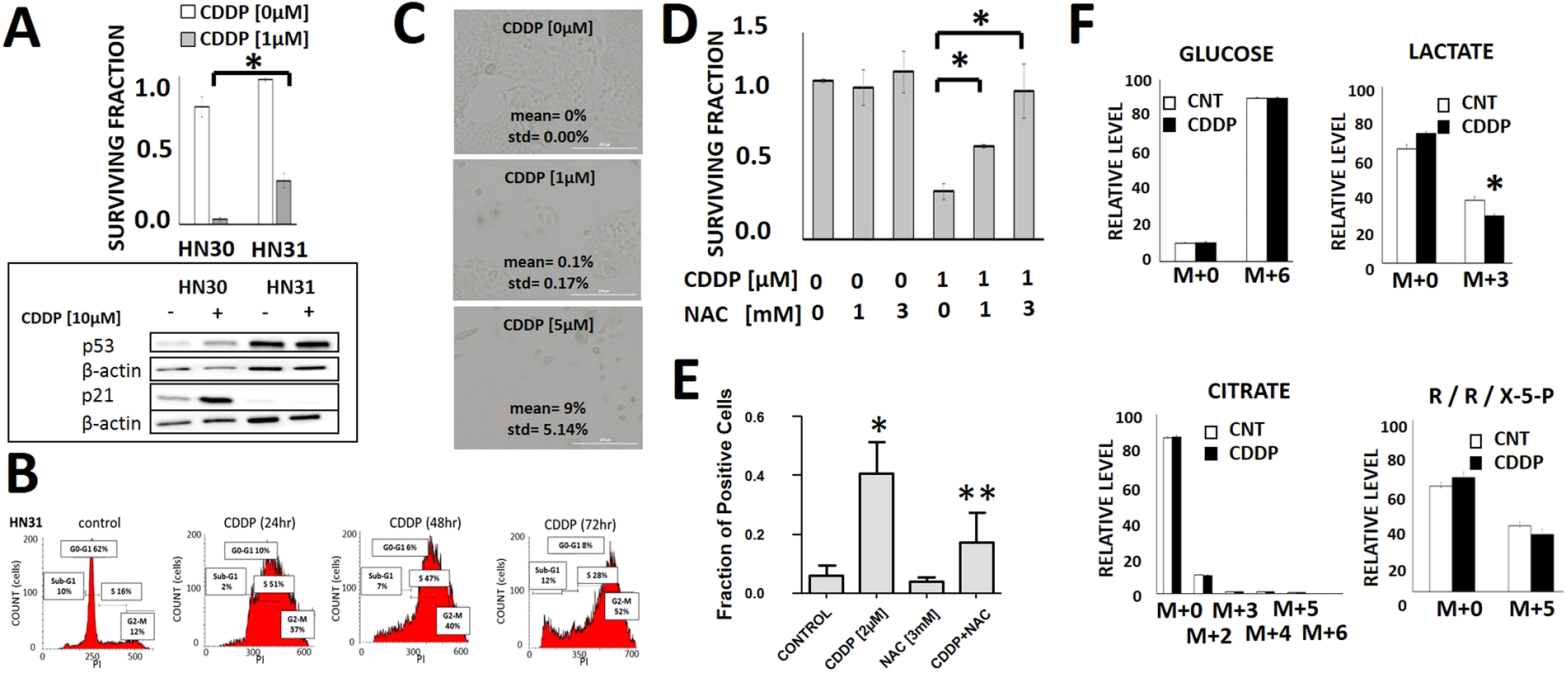
Cisplatin effects on cell proliferation and oxidative stress are independent of *TP53* status. (**A**) HN31 cells were exposed to cisplatin for 24 hours and allowed to form colonies. Surviving fraction was ascertained 10–14 days later and expressed as a function of the control condition. Error bars represent standard deviation; *indicates p < 0.05. Inset demonstrates differential p53 functional status between HN30 and HN31, with CDDP dependent stabilization of p53 in HN30 and activation of p21; p21 activation is absent in HN31 which expresses mutant *TP53*. (**B**) Cells were exposed to CDDP [5 µM] for 24–72 hours and stained with propidium iodide (PI). Flow cytometry was used to ascertain cellular fraction in each phase of the cell cycle. (**C**) Cells were grown at sub-confluence, treated with CDDP for 24 hours and then allowed to recover for 5 days. Cells were then fixed and stained for beta-galactosidase activity. Each experiment was carried out in triplicate. (**D**) Cells were exposed to CDDP in the presence or absence of NAC for 24 hours and allowed to form colonies. Surviving fraction was ascertained 10–14 days later and expressed as a function of the control condition. Error bars represent standard deviation; *Indicates p < 0.05. (**E**) Cells were exposed to CDDP in the presence or absence of NAC and fraction of cells with positive phosphor-γH2AX signal in the nucleus was measured; represented as mean + STD of n = 16 wells/treatment. *Indicates p < 0.05 compared to control condition; **Indicates p < 0.05 compared to CDDP only condition. (**F**) HN31 cells were exposed to 10 mM all carbon (U) ^13^C-labeled glucose for 3 hours in the presence or absence of CDDP [10 µM]. Cells were harvested and metabolite levels (unlabeled- m + 0, labeled- m + (2–6)) were quantitatively measured. Each condition was tested in triplicate. Data are presented as means, with error bars indicating standard error of the mean. (R/R/X-5-P = ribose/ribulose/xylulose-5-phosphate); m + X indicates mass shift of X from unlabeled glucose value. *Indicates change is statistically significant compared to the control condition, p-value < 0.05.

To determine which cisplatin-induced shifts in carbon flux are generalizable we evaluated HN31, an established HNSCC cell line which is isogenic to HN30 with the exception of 2 *TP53* mutations resulting in protein stabilization and loss of function (Fig. 7A inset; Supp. Figure 2)^15,18,24^. In contrast to its isogenic counterpart, HN31 is relatively resistant to CDDP (Fig. 7A), demonstrates high levels of p53 protein consistent with its mutational status and no activation of its canonical down-stream target p21 (Fig. 7A inset; Supp. Figure 2). CDDP effects on HN31 proliferation/survival are driven primarily by accumulation in G2-M (Fig. 7B) and not through increased senescence. Despite this differential mechanism, NAC reverses CDDP effects on HN31 clonogenic survival and γH2AX foci formation indicating that CDDP effects are still driven in part by oxidative stress despite the absence of functional p53 protein (Fig. 7D,E). Cisplatin decreased ^13^C flux from pyruvate into lactate, but in contrast to HN30, shunting into TCA and PPP intermediates was not detectable in HN31 (Fig. 7F).

**Figure 8:**
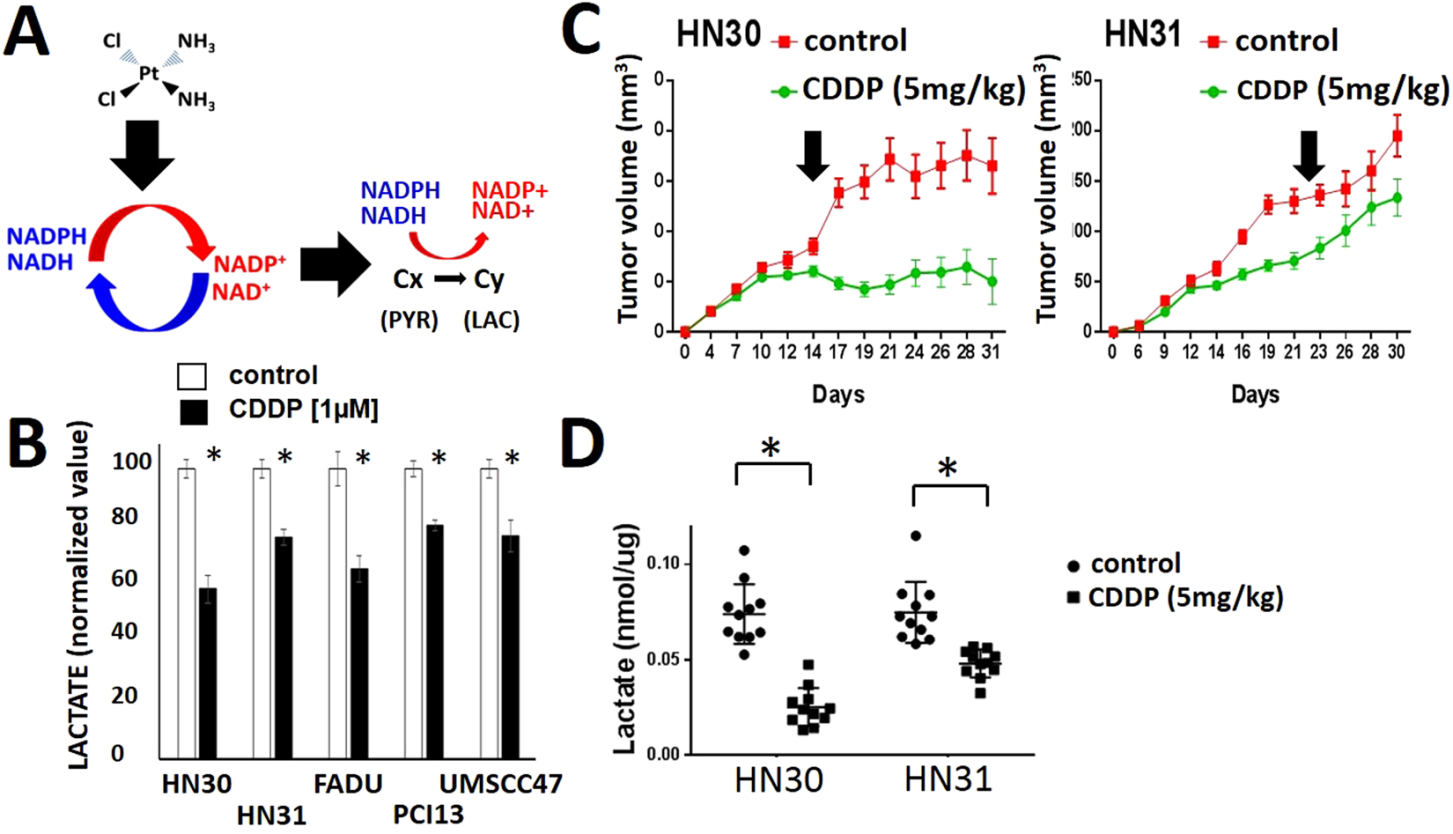
Changes in tumor metabolites correlate with cisplatin anti-tumorigenic effects. (**A**) Biochemical model linking cisplatin induced oxidative stress with carbon flux. (**B**) HNSCC cells were exposed to CDDP for 3 hours and intra-cellular lactate levels were measured biochemically; data are expressed as averages, with error bars presented standard deviation and all data are normalized to the control condition. Each condition was tested at least in triplicate. *Indicates p < 0.05. (**C**,**D**) HN30 and HN31 dual flank tumors were generated by injecting 2 × 10^6^ cells/tumor. Tumors were allowed to grow for 13 days. Mice were treated with a single dose of cisplatin (5 mg/kg). One flank tumor was harvested at 1 hour after treatment and subjected to metabolic interrogation (**B**). *Indicates p < 0.05 compared to control condition. The contralateral tumor was allowed to grow until day 30–31 and tumor size measurements were recorded at regular intervals (**C**).

Since cisplatin-induced changes in pyruvate to lactate flux are conserved across two distinct genomic backgrounds (wt*TP53 vs* mut*TP53*), we sought to determine whether changes in lactate are conserved across multiple HNSCC cell types that reflect the genomic heterogeneity encountered in this disease (Fig. 8A). Since we previously showed a good correlation between *K*_*pl*_ and lactate, and since direct lactate measurements represent a more feasible endpoint for *in vivo* experiments, we measured the impact of CDDP on intra-cellular lactate levels in 5 previously established, described and validated cell lines: HN30, HN31, FADU, PCI-13 and UMSCC-47. Cisplatin triggered at rapid decrease in intra-cellular lactate levels in all 5 cell lines at low µM doses which is consistent with its anti-proliferative effects (Fig. 8B). This does not appear to be due to inactivation of the LDH enzyme since *ex vivo* testing of cellular LDH activity following cisplatin exposure demonstrated preservation of LDH activity (Supp. Figure 3). Exposure of established flank xenografts to a single dose of CDDP resulted in a rapid decrease in tumor lactate levels which correlates with effects on tumor growth delay. Specifically, the relative CDDP resistance demonstrated by HN31 correlated with a smaller change in lactate following acute cisplatin exposure (Fig. 8). This is consistent with differential inhibition of proliferation secondary to LDH inhibition using oxamate (a structural analog of pyruvate) in HN30 and HN31 (Supp. Figure 4A). The addition of oxamate generated a small, but statistically significant increase in cisplatin toxicity in the relatively cisplatin-resistant HN31 cell line (Supp. Figure 4B).

## Discussion

Development of effective treatment strategies for HNSCC requires a robust understanding of tumor cell response to conventional chemotherapeutic agents such as cisplatin. Although DNA damage repair has been extensively studied as a potential driver of tumor response to chemotherapeutic agents, there is little evidence that impaired DNA repair plays an important role in regulating HNSCC response to cisplatin^7,9,25^. Traditionally, cisplatin toxicity has been attributed to DNA binding and generation of single stranded DNA breaks^7,9^. Our data demonstrate that DNA-bound cisplatin represents a very small fraction of total intra-cellular cisplatin and that cisplatin generates robust activation of γH2AX which is consistent with a generalized oxidative stress response in HNSCC. Reversal of oxidative stress abrogates cisplatin toxicity in our model suggesting that cisplatin effects on oxidative stress are an important, if not the critical contributor to its toxicity in the context of HNSCC. Interestingly NAC does decrease DNA-bound cisplatin levels particularly after prolonged exposure. This may be secondary to regeneration of reducing moieties (i.e. sulfhydryl groups) which can then sequester cisplatin prior to DNA binding. The overall relationship between oxidative stress and cisplatin toxicity is independent of functional p53 and is not unique to a specific mechanism of cell death. This is consistent with data generated by the group led by Dr. Douglas R. Spitz using direct manipulation of intracellular oxidative stress^26–29^.

The relative contribution of oxidative stress to cisplatin toxicity is important to establish, because oxidative stress has been shown by us and others to cause compensatory changes in cellular metabolism secondary to decreased availability of reducing equivalents^17^. Reducing equivalents in the form of NADPH and NADH are not only critical for maintaining redox homeostasis, but also critical to basic metabolic reactions that form the backbone of tumor cell energy and biomass generation. As described by Warburg over half a century ago, conversion of glucose into lactate is a nearly universal phenomenon in tumor cells irrespective of oxygen availability^30^. In HNSCC we previously demonstrated that glucose is the predominant requirement for survival and growth and that HNSCC cells demonstrate high rates of glycolytic activity^18,24^. These previous findings are consistent with data generated here using ^13^C-label tracing. Because conversion of pyruvate into lactate is a nearly ubiquitous reaction in HNSCC that requires NADH to proceed in the forward direction, we sought to determine whether redox changes triggered by cisplatin would produce measurable changes in lactate generation. Indeed, cisplatin not only decreases lactate levels in HNSCC tumors within hours of exposure but also decreases the flux through the pyruvate to lactate reaction in HNSCC resulting in decreased ^13^C-label incorporation. Interestingly, decreased flux through this reaction generates compensatory changes in the pentose phosphate pathway (PPP) and the citric acid cycle (TCA) as demonstrated through increased ^13^C incorporation into metabolites in these pathways following cisplatin administration. Since carbon shunting appears to increase with prolonged cisplatin exposure, it may represent an attempt to adapt to oxidative stress and re-equilibrate the redox state of the cell.

Cisplatin-induced changes in carbon flux can provide information potentially useful for prediction of treatment response. As such, we sought to determine which pathway and/or enzymatic step was consistently perturbed by cisplatin exposure. Our data indicate that significant shunting of carbon occurs in HN30 cells, resulting in increased flux through PPP and TCA intermediates, but not in HN31 cells. Despite their nearly identical molecular background, we previously showed that loss of p53 function through mutation increases lactate production in HN31 and decreases reserve mitochondrial capacity^18,24^. Our present data confirm this differential metabolic phenotype using a complementary approach. They also demonstrate that lactate is the only common carbon intermediate that is consistently decreased in response to cisplatin-induced oxidative stress across multiple HNSCC cell lines of varying genomic backgrounds. Measurements of acute lactate changes in a preclinical model of HNSCC are consistent with cisplatin effects on tumor growth delay and the effects of direct LDH inhibition appear consistent with our metabolomics data.

Baseline tumor and cellular lactate levels have been inconsistently associated with tumor characteristics and treatment response^31–33^. In large part, this is because *baseline* lactate levels reflect the extent to which tumors behave as predicted by the Warburg effect. In contrast, the *acute* changes in lactate demonstrated here, should represent a nearly universal phenomenon as long as the tumors in question express the common isoforms of lactate dehydrogenase as HNSCC tumors have been shown to do. Indeed, acute changes in cellular lactate are consistent across the HNSCC cell lines utilized here and are preserved in the *in vivo* model. It remains to be seen whether the magnitude of changes in lactate is sufficiently robust to predict relative sensitivity and/or resistance to cisplatin, and this is the focus of our planned studies using hyperpolarized-magnetic resonance imaging (HP-MRI)^33–44^. Using HP tracers, it is now possible to interrogate solid tumor metabolism with increasing temporal, spatial and chemical resolution. What is urgently needed is an understanding of which metabolic targets may be suitable for interrogation to assess tumor response to conventional chemotherapeutic agents. In this study we begin to address this question in the context of cisplatin and identify acute changes in lactate generation as a potentially universal biomarker of treatment response in HNSCC. Whether this type of metabolomics analysis can also be used to identify metabolic-based cisplatin and radiation sensitization strategies remains to be determined, as efforts at metabolic targeting have showed variable effectiveness to date in the preclinical and clinical setting^18,23,24,26,45–49^.

## Electronic supplementary material

Supplemental Figures and Legends
