# Supplementary material for: Cisplatin generates oxidative stress which is accompanied by rapid shifts in central carbon metabolism
Source: Sci Rep. 2018 Mar 9;8:4306. doi: 10.1038/s41598-018-22640-y (PMC5844883; doi:10.1038/s41598-018-22640-y)
Supplement: Supplementary file 1 — Supplemental Figures and Legends [file 41598_2018_22640_MOESM1_ESM.pdf]

Cisplatin generates oxidative stress which is accompanied by rapid shifts in central carbon metabolism

Wangie Yu [1*], Yunyun Chen [2*], Julien Dubrulle [6], Fabio Stossi [3,6], Vasanta Putluri [4], Arun Sreekumar [3], Nagireddy Putluri [3], Dodge Baluya [5],Stephen Y. Lai [2,7]§, Vlad C. Sandulache [1]§

Author's Affiliations:

1- Bobby R. Alford Department of Otolaryngology Head and Neck Surgery, Baylor College of Medicine, Houston, TX

2- Department of Head and Neck Surgery, University of Texas MD Anderson Cancer Center, Houston, TX

3- Department of Molecular and Cellular Biology, Baylor College of Medicine, Houston, TX

4- Advanced Technology Core, Dan Duncan Cancer Center, Baylor College of Medicine, Houston, TX

5- Chemical Imaging Research Core, University of Texas MD Anderson Cancer Center, Houston, TX

6 – Integrated Microscopy Core, Advanced Technology Cores, Baylor College of Medicine, Houston, TX

7- Department of Molecular and Cellular Oncology, University of Texas MD Anderson Cancer Center, Houston, TX

*These authors contributed equally to this work.

§Both senior authors contributed equally to this work.

SUPPLEMENTAL FIGURE 1

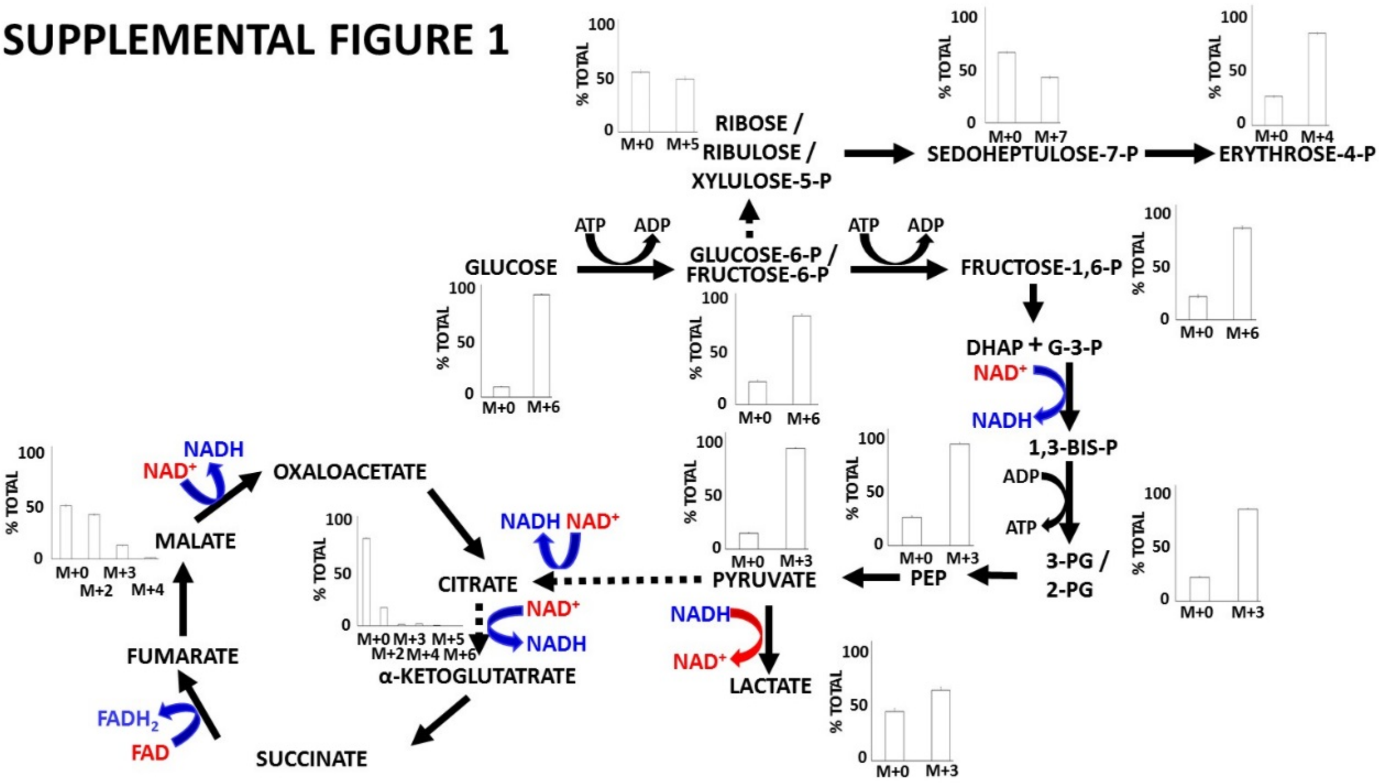

Supplemental Figure 1: HNSCC cells exhibit preferential conversion of glucose into lactate under aerobic conditions. HN30 cells were exposed to 10mM all carbon (C6) ^{13}C-labeled glucose for 3 hours. Cells were harvested and metabolite levels (unlabeled- m+0, labeled- m+X) were quantitatively measured. Reactions involving energy (ATP) generation and consumption are labeled along with reactions which generate reducing equivalents in the form of NADH and FADH2. (P= phosphate, DHAP= dihydroxyacetone phosphate, G-3-P= glyceraldehyde-3-phosphate, 3/2-PG= 3/2 phosphoglycerate, 1-3-Bis-P= 1,3, bisphosphoglycerate, PEP= phosphoenolpyruvate); m+X indicates mass shift of X from unlabeled glucose value indicating incorporation of ^{13}C.

SUPPLEMENTAL FIGURE 2

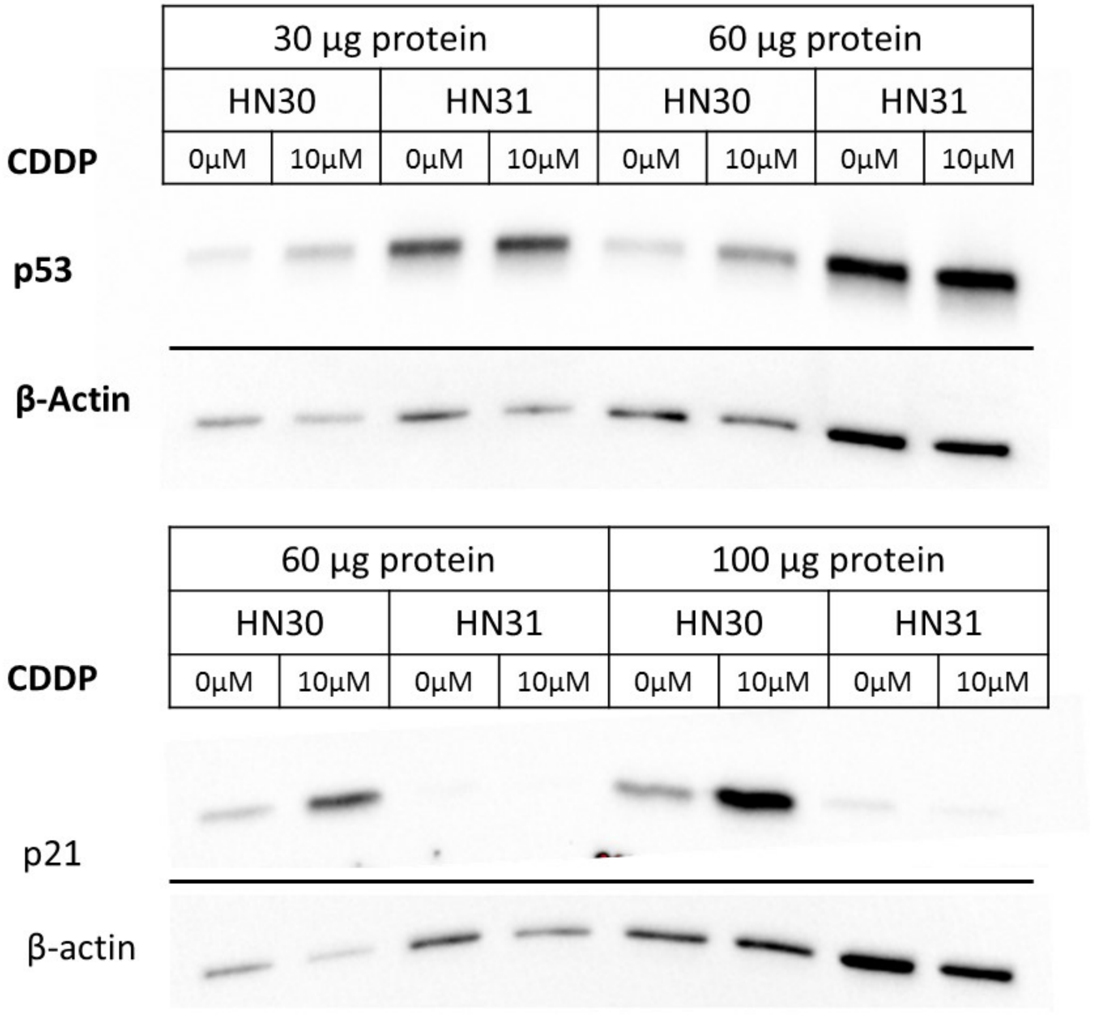

Supplemental Figure 2: Differential p53 status and activity in HNSCC cells. Cells were exposed to CDDP for 24 hours. Cells lysates were then subjected to Western blotting using antibodies targeting p53 (total) and p21 (total). Raw blots are shown above, along with experimental conditions and the total amount of protein loaded for each condition. Images represent the full blots but membranes were cut before incubation and exposure to facilitate staining for individual proteins. The horizontal black lines delineate

individual proteins as they were probed for separately. All tested conditions were ran together for each individual protein.

SUPPLEMENTAL TABLE 1

			CDDP [µM]	CDDP [µM]	CDDP [µM]
**Cell line**	**Time (hr)**	**control**	**1**	**5**	**10**
**HN30**	**1**	**1**	***1.05***	***1.01***	***1.01***
	**3**	**1**	***1.08***	***1.05***	***1.05***
**HN31**	**1**	**1**	1.08	1.00	***0.94***
	**3**	**1**	***1.03***	***1.03***	0.99

Supplemental Table 1. Cisplatin effects on intra-cellular ATP levels. HN30 and HN31 were exposed to increasing doses of cisplatin (CDDP) for 1 or 3 hours and intra-cellular ATP levels were measured. ATP levels were compared to the control condition. Bold italicized values are statistically significant by Student's t-test, with a p-value of <0.05. Each condition was tested in 7 replicates and each experiment was performed at least twice.

SUPPLEMENTAL FIGURE 3

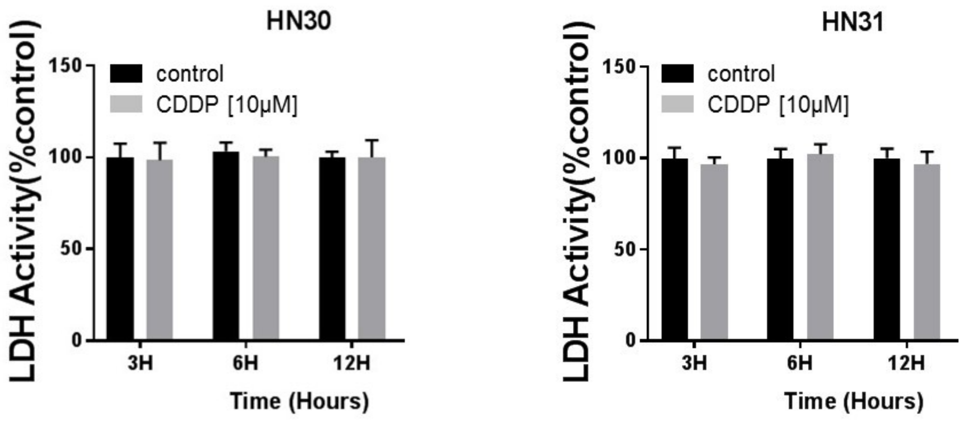

Supplemental Figure 3. LDH activity is not directly impaired by cisplatin. HN30 and HN31 were exposed to increasing doses of cisplatin (CDDP) for 3, 6 or 12 hours and LDH activity was measured in the cellular lysates. LDH activity was normalized to total cellular protein and data are presented as a fraction of the control condition LDH activity value. Each condition was tested in triplicate and each experiment was performed at least twice. Data are presented as means, with error bars demonstrating standard deviation.

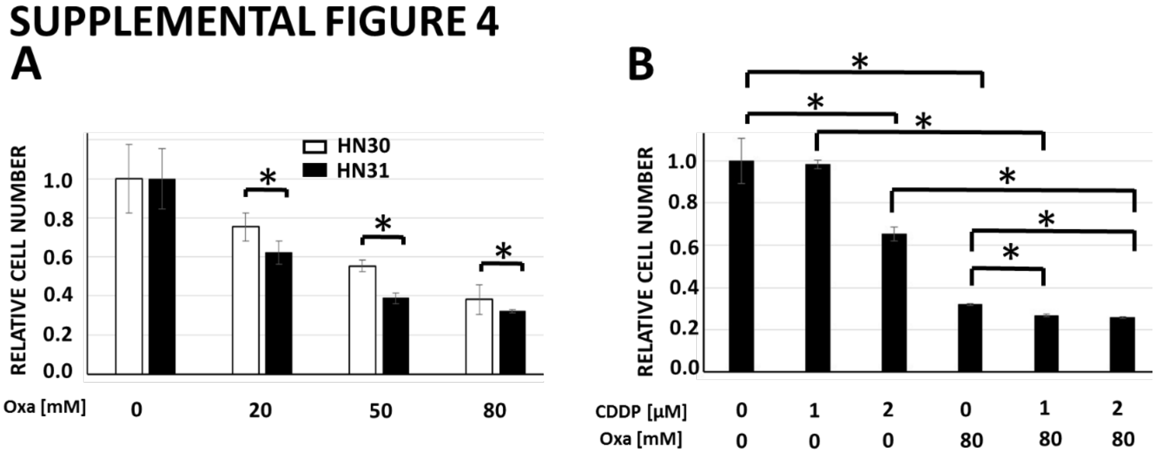

Supplemental Figure 4. LDH inhibition decreases HNSCC proliferation. A) HN30 and HN31 cells were exposed to increasing doses of oxamate for 72 hours. Relative cell numbers were calculated at the end of the experimental period and data are expressed as a fraction of the control condition. B) HN31 cells were exposed to oxamate in the presence or absence of cisplatin (CDDP) for 72 hours. Relative cell numbers were calculated at the end of the experimental period and data are expressed as a fraction of the control condition. Each condition was tested using 8 replicates and each experiment was performed at least twice. Data are presented as means, with error bars demonstrating standard deviation. * indicates p-value <0.05 using the Student's t-test.
